# The relationship between total and phosphorylated STAT1 and STAT3 tumour cell expression, components of tumour microenvironment and survival in patients with invasive ductal breast cancer

**DOI:** 10.18632/oncotarget.12730

**Published:** 2016-10-18

**Authors:** Fadia J.A. Gujam, Donald C. McMillan, Joanne Edwards

**Affiliations:** ^1^ Academic Unit of Surgery, College of Medical, Veterinary and Life Sciences-University of Glasgow, Royal Infirmary, Glasgow, Scotland; ^2^ Unit of Experimental Therapeutics, Institute of Cancer, College of Medical, Veterinary and Life Sciences-University of Glasgow, Wolfson Wohl Cancer Research Centre, Glasgow, Scotland

**Keywords:** breast cancer, STAT1, STAT3, tumour microenvironment and survival

## Abstract

The aim of the present study was to examine the relationship between tumour cell expression of total and phosphorylated STAT1 (ph-STAT1) and STAT3 (ph-STAT-3), components of tumour microenvironment and survival in patients with invasive ductal breast cancer.

Immunohistochemical analysis of total and ph-STAT1, and STAT3 were performed on tissue microarray of 384 breast cancer specimens. Tumour cell expression of STAT1 and STAT3 at both cytoplasmic and nuclear locations were combined and identified as STAT1/STAT3 tumour cell expression. These results were related to cancer specific survival (CSS) and phenotypic features of the tumour and the host.

High ph-STAT1 and ph-STAT3 tumour cell expression were associated with increased ER (both *P*≤0.001) and PR (both *P* <0.05), reduced tumour grade (*P*=0.015 and *P*<0.001 respectively) and necrosis (both *P*=0.001). Ph-STAT1 was associated with increased general inflammatory infiltrate (*P*=0.007) and ph-STAT3 was associated with lower CD4+ infiltration (*P*=0.024). In multivariate survival analysis, only high ph-STAT3 tumour cell expression was a predictor of improved CSS (*P*=0.010) independent of other tumour and host-based factors.

STAT1 and STAT3 tumour cell expression appeared to be an important determinant of favourable outcome in patients with invasive ductal breast cancer. The present results suggest that STAT1 and STAT3 may affect disease outcome through direct impact on tumour cells, counteracting aggressive tumour features, as well as interaction with the surrounding microenvironment.

## INTRODUCTION

Breast cancer is the most frequent malignancy in women, accounting for 25% of all female cancers, with more than a million women diagnosed annually. It is also the leading cause of cancer death in women with >500,000 deaths in 2012 worldwide [1]. Therefore, it is clear that there is a need to identify characteristics applicable to both the tumour and the host to not only guide prognosis, but also identify potential therapies.

Components of tumour microenvironment, including tumour stroma and tumour inflammatory cell infiltrates are now recognised to play a key role in cancer progression and survival, and represent interactions between the tumour and the host [2, 3]. However, the underlying mechanism of the interaction between the different components of tumour microenvironment is not fully understood. Cross-talk between signaling pathways determine how a cell integrates the environmental signals received, ultimately translating them in transcriptional regulation of specific sets of genes [4]. Signal transducers and activators of transcription family (STATs) have been recognized to act downstream of cytokine and growth factor receptors [4, 5], and may therefore play a central role in determining the phenotypic characteristics of the tumour.

The IL-6/Janus-activated kinase can trigger tyrosine phosphorylation of both STAT1 and STAT3 through homo- or hetero-dimerization of the signal transduction subunit gp130 [6]. STATs detect a variety of signals at the cell membrane and transduce them to the nucleus directly affecting gene regulation of cell growth, survival, differentiation, and motility. STAT1 is a central mediator of both type I and type II interferon (IFN) [7, 8], however both IFNs can also activate STAT3 (6).

STAT1 and STAT3 have a complex interaction with both tumour cells and the tumour microenvironment including immune infiltrates such that STAT1 and STAT3 are thought to play opposite roles in tumorigenesis, regulating distinct gene signatures [9]. STAT1 has been considered as a growth suppressor based on its role as a pro-apoptotic and anti-proliferative molecule [4, 5]. STAT3 is well established as a key factor in mammary epithelial cell growth and differentiation behaving as an oncogene [10] and also, is essential in mammary gland epithelial cell apoptosis and involution [11, 12]. Furthermore, studies in STAT-deficient cells/animals have revealed the existence of reciprocal STAT1 to STAT3 regulatory mechanisms that represent cross-regulation between the two molecules [9] such that STAT3 gene inactivation results in increased and prolonged phosphorylation of STAT1 in response to gp130 cytokines [6, 9].

Despite the fact that several experimental studies suggest that STAT1 and STAT3 play a critical role in breast cancer tumorigenesis, the prognostic value of these proteins in patients with breast cancer remains unclear. Five studies have examined the prognostic value of STAT1 in breast cancer, using either total STAT1 or phosphorylated STAT1 (ph-STAT1) (Table 1). An initial analysis by Widschwendter et al using Western blotting and DNA binding technique, reported an independent association between high ph-STAT1 and improved overall and cancer specific survival (CSS) [13]. In contrast, immunohistochemistry (IHC) of ph-STAT1, found that ph-STAT1 in premenopausal women was associated with poor overall survival, but not in postmenopausal women. However, co-expression of ph-STAT1 with ER or PR was associated with longer CSS in postmenopausal women [14]. Studies measuring total STAT1 have also reported conflicting results. High total STAT1 was not associated with outcome in two studies [15, 16] and was a significant predictor of worse survival in one study [17] (Table 1).

**Table 1:** Studies on the prognostic significance of STAT1 and STAT3 in breast cancer

References	Patients	Sample size	Follow-up	Protein examined	Association with outcome
**STAT1 studies**					
Widschwendter et al., 2002	N/S	53	6.8	ph-STAT1	associated with improved overall and CSS (multivariate analysis)
Sheen Chen et al., 2007	N/S	102	5.8	total STAT1	no association with overall survival
Charpin et al., 2009	N/S	924	6.5	total STAT1	associated with reduced CSS
Magkou et al., 2012	Premenopausal/postmenopausal	165	7.5	ph-STAT1	in premenopausal women: associated with poor OS (univariate analysis)
					in postmenopausal women: co-expression with ER/or PR was associated with improved CSS (univariate analysis)
Huang et al., 2014	N/S	546	15	total STAT1	no significant association with CSS
**STAT3 studies**					
Widschwendter et al., 2002	N/S	53	6.8	ph-STAT3	no association with survival
Dolled-Filhart et al., 2003	LN -ve	255	5 & 20	total STAT3	associated with improved OS
Yamashita et al., 2006	N/S	506	7.5	ph-STAT3	no association with OS and CSS
Sheen-Chen et al., 2008	N/S	102	5	total STAT3	associated with reduced OS
Charpin et al., 2009	N/S	924	6.5	ph-STAT3	associated with reduced CSS
Sato et al., 2011	all, LN-ve/ LN+ve, low & high grade	721	>10	total STAT3	associated with improved OS in patients with low grade tumours (univariate analysis)
Sonnenblick et al., 2012	LN +ve	125	5 & 10	ph-STAT3	associated with improved OS
Sonnenblick et al., 2013	N/S	375	10	ph-STAT3	associated with improved OS in patients treated with adjuvant chemotherapy
Huang et al., 2014	N/S	546	15	total STAT3	associated with improved CSS (univariate analysis)
Aleskandarany et al., 2016	N/S	1270	N/A	Ph-STAT3	associated with improved CSS (multivariate analysis)

N/S: not specified invasive breast cancer, LN: lymph node, ph-STAT1 tyrosine phosphorylated STAT1, ph-STAT3: tyrosine phosphorylated STAT3, CSS: cancer specific survival. OS: overall survival, Follow-up in years. N/A: not available.

Ten studies have examined the prognostic value of STAT3 in breast cancer, using either total STAT3 or phosphorylated STAT3 (ph-STAT3) (Table 1). High total STAT3 was significantly associated with improved outcome in three studies [16, 18, 19], and with poor outcome in one study [20]. Ph-STAT3 expression was not associated with breast cancer survival in two studies [13, 21], and was associated with improved survival in large cohort of patients [22], patients with lymph node positive tumours [23], and patients treated with adjuvant chemotherapy [24]. In contrast, ph-STAT3 was a significant predictor of worse survival in one study [17] (Table 1). Therefore, given that clinical trials evaluating Il-6/JAK/ STAT inhibitors in breast cancer patients are under way [25], it would be important to determine the role of STAT1 and STAT3 in this disease.

Also, commensurate with their role in regulating cytokine-dependent inflammation and immunity, the relationship between STAT1 and STAT3 and components of tumour microenvironment is unclear. Therefore, the aim of the present study was to examine the relationship between total and phosphorylated STAT1 and STAT3 tumour cell expression, components of the tumour microenvironment and survival in a mature cohort of patients with invasive ductal breast cancer.

## RESULTS

**Figure 1:**
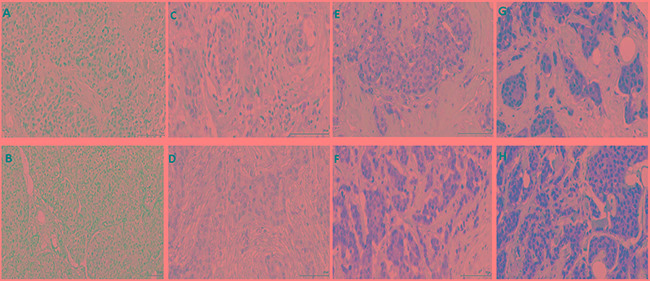
Sections of invasive ductal beast carcinomas showing IHC expression levels of ph-STAT1 (first row) and ph-STAT3 (second row) No appreciable expression was detected in the negative controls of ph-STAT1 **A.** and ph-STAT3 **B**. **C-H.** show the staining intensity of the STAT1 and STAT3 expression as low (C and D), moderate (E and F), and strong (G and H). Original magnification, 20×. Scale bars = 100 μm (A-F), 10 μm (G and H).

Total and ph-STAT1 and STAT3 expression in tumour cells were quantified using the weighted histoscore, taking the staining intensity and percentage into account (material and methods). The IHC staining of total and ph-STAT1 and STAT3 was homogenous in both the cytoplasm and nuclei of tumour cells, which is consistent with previous reports [22]. The staining was also observed in the surrounding stromal cells (fibroblasts and infiltrating inflammatory cells) with variable degrees of positivity. Figure 1 displays images representative of STAT1 and STAT3 IHC staining.

The histoscore of total STAT1 expression ranged from 0-200 within the cytoplasm and from 0-220 within the nucleus, with cytoplasmic and nuclear expression in 270 patients (70%) and 268 patients (70%) respectively. The histoscore for ph-STAT1 expression ranged from 0-190 within the cytoplasm and from 0-225 within the nucleus, with cytoplasmic and nuclear expression in 350 patients (91%) and 374 patients (97%) respectively. Total STAT1 cytoplasmic expression was not correlated with ph-STAT1 nuclear expression (*P*=0.421). Expression of total STAT1 and ph-STAT1 within the nucleus correlated strongly with their expression within the cytoplasm (all *P*<0.001).

The histoscore of total STAT3 expression ranged from 0-280 within the cytoplasm and from 0-293 within the nucleus, with cytoplasmic and nuclear expression in 375 patients (98%). The histoscore of ph-STAT3 expression ranged from 0-150 within the cytoplasm and from 0-250 within the nucleus, with cytoplasmic and nuclear expression in 359 patients (93%) and 376 patients (98%) respectively. Total STAT3 tumour cell expression correlated strongly with ph-STAT3 tumour cell expression (*P*<0.001). Expression of total STAT3 and ph-STAT3 within the nucleus correlated strongly with their expression within the cytoplasm (all *P*<0.001).

The clinical and pathological characteristics of patients with invasive ductal breast cancer are shown in Table 2. The majority of patients aged 50 years or older (70%), had a tumour size ≤ 2 cm (61%), grade III carcinoma (43%) with negative axillary lymph node involvement (54%). The majority had ER positive tumours (68%), PR positive tumours (60%) and Her-2 negative tumours (79%), with high grade tumour necrosis (53%). 241 (63%) patients had mastectomy with radiotherapy, 194 (51%) patients received only hormonal therapy, 90 (23%) received only chemotherapy, and 70 (18%) received both. 174 (45%) of patients had Luminal A tumours, 92 (24%) had Luminal B tumours, 30 (8%) had Her-2 positive tumours and 68 (18%) had Triple negative tumours.

**Table 2:** The clinicopathological characteristics of patients with invasive ductal

Clinicopathological characteristics	Patients, n (%)
Age (≤50/ >50 years)	116(30%)/268(70%)
Size (≤20/ 21-50/ >50 mm)	233(61%)/142(37%)/9(2%)
Grade (I / II / III)	71(19%)/147(38%)/166(43%)
Involved lymph node (−ve/+ve)	209(54%)/172(45%)*
ER status (no/yes)	116(30%)/268(68%)
PR status (no/yes)	152(40%)/230(60%)*
Her-2 status (no/ yes)	305(79%)/70(18%)*
Lymphaic vessel invasion (no/yes)	254(66%)/130(34%)
Blood vessel invasion (no/yes)	340(88%)/44(12%)
Tumour necrosis (low/high)	183(48%)/201(52%)
Klintrup–Mäkinen grade (low/high)	272(71%)/112(29%)
CD68+ (low/moderate/high)	116(30%)/129(34%)/124(32%)*
CD4+ (low/moderate/high)	160(42%)/75(20%)/136(35%)*
CD8+ (low/moderate/high)	124(32%)/119(31%)/128(33%)*
CD138+(low/moderate/high)	203(53%)/45(12%)/122(32%)*
Tumour stroma percentage (low/high)	264(69%)/120(31%)
Tumour budding (low/high)	250(65%)/134(35%)
Locoregional treatment (lumpectomy + radiotherapy/mastectomy +radiotherapy)	143(37%)/241(63%)
Systemic treatment (hormonal/hormonal+ chemotherapy/chemotherapy/ none)	194(51%)/70(18%)/90(23)/24(6%)*
Recurrence status (no/yes)	285(74%)/95(25%)*
Alive/cancer death/non cancer death	228(59%)/82(22%)/74(19%)

* Number of patients when incomplete data available.

The relationship between ph-STAT1 and ph-STAT3 tumour cell expression and clinicopathological characteristics was shown in Table 3. Ph-STAT1 tumour cell expression was not associated with patient age, tumour size, Her-2 status, or the presence of lymphatic (LVI) and blood (BVI) vessel invasion. High ph-STAT1 tumour cell expression was positively associated with ER status (*P*=0.001), PR status (*P*=0.048), and negatively with increased tumour grade (*P*=0.015). Similarly, ph-STAT3 tumour cell expression was not associated with patient age, tumour size and Her-2 status, though borderline significant associations with reduced LVI (*P*=0.055) and BVI (*P*=0.052) were observed. High ph-STAT3 tumour cell expression was positively associated with ER status (*P*<0.001), PR status (*P*=0.015) and negatively with increased tumour grade (*P*<0.001).

**Table 3:** The relationship between ph-STAT1 and ph-STAT3 tumour cell expression and clinicopathological characteristics (n=384)

	Ph-STAT1 tumour cell expression				Ph-STAT3 tumour cell expression			
	low	moderate	high	*P* value	low	moderate	high	*P* value
	n=127, 33%	n=136, 35%	n=121, 32%		n=154, 40%	n=121, 32%	n=109, 28%	
Age (≤50/ >50 years)	35/92	38/98	43/78	0.175	46/108	40/81	30/79	0.744
Size (≤20/21-50/>50 mm)	73/48/6	88/47/1	72/47/2	0.444	91/58/5	73/46/2	69/38/2	0.402
Grade (I / II / III)	17/43/67	29/53/54	25/51/45	0.015	14/60/80	25/47/49	32/40/37	<0.001
Lymph node status (−ve/+ve)	63/62	78/57	68/53	0.357	76/76	66/55	67/41	0.057
ER status (no/yes)	50/77	41/95	25/96	0.001	66/88	32/89	18/91	<0.001
PR status (no/yes)	57/70	56/79	39/81	0.048	70/83	49/72	33/75	0.015
Her-2 status (no/ yes)	99/26	102/29	104/15	0.105	120/31	94/25	91/14	0.173
Tumour necrosis (low/high)	45/82	69/67	69/52	0.001	60/94	57/64	66/43	0.001
Lymphatic vessel invasion (no/yes)	85/42	89/47	80/41	0.890	96/58	77/44	81/28	0.052
Blood vessel invasion (no/yes)	109/18	123/13	108/13	0.390	133/21	104/17	103/6	0.055
Klintrup–Mäkinen grade (low/high)	97/30	98/38	77/44	0.007	108/46	81/40	83/26	0.347
CD68+ (low/moderate/high)	40/47/33	40/39/54	36/43/37	0.514	49/53/45	42/33/42	25/43/37	0.183
CD4+ (low/moderate/high)	47/30/44	55/28/51	58/17/41	0.297	57/30/61	51/18/48	52/27/27	0.024
CD8+ (low/moderate/high)	46/38/37	39/51/44	39/30/47	0.179	52/43/53	36/39/42	36/37/33	0.785
CD138+(low/moderate/high)	65/14/42	74/14/45	64/17/35	0.613	90/15/42	61/12/44	52/18/36	0.109
Tumour strtoma percentage (low/high)	83/44	93/43	88/33	0.212	99/55	91/30	74/35	0.426
Tumour budding (low/high)	88/39	79/57	83/38	0.884	96/58	76/45	78/31	0.140
Locoregional treatment (lumpectomy+radiotherapy/mastectomy +radiotherapy)	47/80	52/84	44/77	0.920	55/99	43/78	45/64	0.385
Systemic treatment (hormonal/hormonal+ chemotherapy/chemotherapy/ none)	58/23/37/8	70/27/28/10	66/20/25/6	0.102	72/26/48/7	57/24/30/7	65/20/12/10	0.060
Recurrence status (no/yes)	86/40	99/37	100/18	0.003	105/49	88/32	92/14	0.001

Within the tumour microenvironment, high ph-STAT1 tumour cell expression was not associated with tumour stroma percentage (TSP) and tumour budding. High ph-STAT1 tumour cell expression was negatively associated with tumour necrosis (*P*=0.001), and was positively associated with the generalised inflammatory infiltrate as measured using Klintrup–Mäkinen (K-M) grade (*P*=0.007). Similarly, high ph-STAT3 tumour cell expression was not associated with TSP and tumour budding. High ph-STAT3 tumour cell expression was negatively associated with tumour necrosis (*P*=0.001) and cellular inflammatory infiltrate as measured using CD4+ helper T-lymphocytes (*P*=0.024). High ph-STAT1 and ph-STAT3 tumour cell expression were also significantly associated with reduced tumour recurrence (*P*=0.003 and *P*=0.001 respectively).

**Figure 2:**
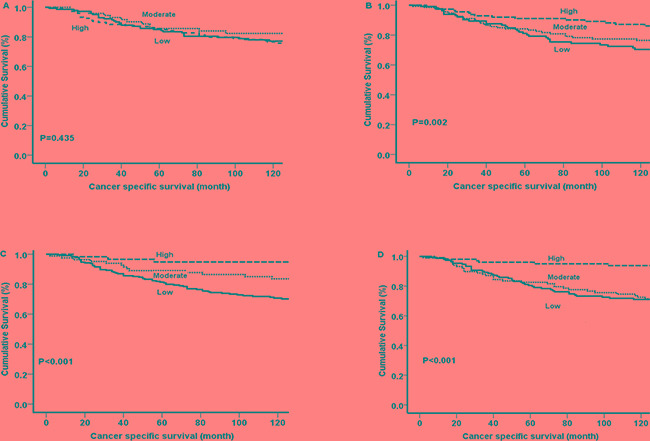
Kaplan-Meier survival curves (Log rank) of cancer specific survival **A.** Total STAT1 tumour cell expression and **B.** Ph-STAT1 tumour cell expression. **C.** Total STAT3 tumour cell expression and **D.** Ph-STAT3 tumour cell expression.

The median follow-up of survivors was 148 months, with 82 cancer-associated deaths and 74 non-cancer deaths. The relationship between total and ph-STAT1 tumour cell expression and CSS using Kaplan-Meier log rank test was examined (Figure 2). The total STAT1 tumour cell expression was not associated with CSS (*P*=0.435) (Figure 2A). High ph-STAT1 tumour cell expression was associated with improved CSS compared to low tumour cell expression (*P*=0.002) (Figure 2B). The mean survival of patients with low ph-STAT1 tumour cell expression was 140 months (95% CI 129-151 months) and 10-year survival rate was 68%, whereas the mean survival of patients with high expression was 160 months (95% CI 152-169 months) and 10-year survival rate was 84%.

The relationship between total and ph-STAT3 tumour cell expression and CSS using Kaplan-Meier log rank test was subsequently examined (Figure 2). Total STAT3 tumour cell expression was associated with CSS (*P*<0.001) (Figure 2C). High ph-STAT3 tumour cell expression was associated with improved CSS compared to patients with low tumour cell expression (*P*<0.001) (Figure 2D). The mean survival of patients with low expression was 139 months (95% CI 129-149 months) and 10-year survival was 62%, whereas the mean survival of patients with high expression was 170 months (95% CI 163-176 months) and 10-year survival was 80%.

The relationship between ph-STAT1 and ph-STAT3 tumour cell expression, clinicopathological characteristics, and CSS is presented in Table 4. In univariate analysis, both high ph-STAT1 (*P*=0.002) and ph-STAT3 (*P*<0.001) tumour cell expression were associated with improved CSS. In multivariate analysis, high ph-STAT1 tumour cell expression was not independently associated with CSS (*P*=0.193). In contrast, high ph-STAT3 tumour cell expression was independently associated with improved CSS (HR 0.64, 95% CI 0.64-0.90, *P*=0.010) independent of other variables, including nodal status, tumour necrosis, LVI, BVI, CD8+ T-lymphocyte infiltrate, CD138+ plasma cell infiltrate, and tumour budding (Table 4).

**Table 4:** The relationship between clinicopathological characteristics, ph-STAT1 and ph-STAT3 tumour cell expression and cancer specific survival in patients with invasive ductal breast cancer (n=384)

	Univariate analysis		Multivariate analysis	
	HR (95% CI)	*P*-value	HR (95% CI)	*P*-value
Age (≤50/ >50 years)	1.14(0.70-1.85)	0.604		
Size (≤20/ 21-50/ >50 mm)	2.21(1.52-3.23)	<0.001		0.475
Grade (I / II / III)	1.89(1.37-2.63)	<0.001		0.254
Involved lymph node (−ve/+ve)	3.85(2.37-6.24)	<0.001	1.90(1.10-3.29)	0.021
ER status (no/yes)	0.54(0.35-0.84)	0.006		0.141
PR status (no/yes)	0.58(0.38-0.90)	0.015		0.181
Her-2 status (no/ yes)	2.05(1.26-3.32)	0.004		0.272
Tumour necrosis (low/high)	5.87(3.26-10.67)	<0.001	4.42(2.31-8.45)	<0.001
Lymphatic vessel invasion (no/yes)	4.08(2.61-6.37)	<0.001	1.94(1.13-3.31)	0.015
Blood vessel invasion (no/yes)	3.28(1.98-5.43)	<0.001	1.79(1.02-3.14)	0.044
Klintrup–Mäkinen grade (low/high)	1.47(0.93-2.23)	0.099		0.526
CD68+ (low/moderate/high)	0.79(0.59-1.02)	0.069		0.101
CD4+ (low/moderate/high)	0.99(0.78-1.26)	0.982		
CD8+ (low/moderate/high)	0.62(0.47-0.82)	<0.001	0.58(0.42-0.80)	0.003
CD138+(low/moderate/high)	1.34(1.06-1.69)	0.014	1.65(1.25-2.18)	<0.001
Tumour stroma percentage (low/high)	2.17(1.40-3.35)	<0.001		0.096
Tumour budding (low/high)	2.46(1.59-3.78)	<0.001	1.88(1.17-3.03)	0.009
Ph-STAT1 tumour cell expression (low/moderate/high)	0.65(0.49-0.86)	0.002		0.193
Ph–STAT3 tumour cell expression (low/moderate/high)	0.54(0.40-0.74)	<0.001	0.64(0.64-0.90)	0.010
Locoregional treatment (lumpectomy +radiotherapy/mastectomy+radiotherapy)	2.62(1.55-4.42)	0.001		0.054
systemic treatment (hormonal/hormonal +chemotherapy/chemotherapy/none)	1.26(1.02-1.55)	0.020		0.408

Due to the strong association observed between both ph-STAT1 and ph-STAT3 and tumour necrosis, the relationship between ph-STAT1 and ph-STAT3 tumour cell expression with CSS in patients with high tumour necrosis was subsequently examined (Table 5). In univariate analysis, high ph-STAT3 but not ph-STAT1 tumour cell expression was significantly associated with improved CSS. In multivariate analysis, high ph-STAT3 tumour cell expression was significantly associated with improved CSS (HR 0.69, 95% CI 0.51-0.95, *P*=0.030) independent of LVI, BVI, CD68+ macrophage infiltrate, CD8+ T-lymphocyte infiltrate, tumour budding and locoregional treatment (Table 5).

**Table 5:** The relationship between clinicopathological characteristics, ph-STAT1 and ph-STAT3 tumour cell expression and cancer specific survival in patients with high grade necrosis (n=201)

	Univariate analysis		Multivariate analysis	
	HR (95% CI)	*P*-value	HR (95% CI)	*P*-value
Age (≤50/ >50 years)	1.15(0.68-1.94)	0.604		
Size (≤20/ 21-50/ >50 mm)	1.66(1.09-2.50)	0.016		0.330
Grade (I / II / III)	1.17(0.78-1.73)	0.452		
Involved lymph node (−ve/+ve)	2.36(1.39-4.03)	0.002		0.156
ER status (no/yes)	0.77(0.48-1.24)	0.303		
PR status (no/yes)	0.78(0.49-1.27)	0.326		
Her-2 status (no/ yes)	1.19(0.71-2.02)	0.503		
Lymphatic vessel invasion (no/yes)	3.28(1.98-5.44)	<0.001	2.53(1.45-4.44)	0.001
Blood vessel invasion (no/yes)	2.78(1.62-4.77)	<0.001	2.03(1.14-3.59)	0.015
CD68+ (low/moderate/high)	0.68(0.51-0.89)	0.007	0.65(0.46-0.91)	0.013
CD4+ (low/moderate/high)	0.78(0.59-1.02)	0.064		0.425
CD8+ (low/moderate/high)	0.51(0.38-0.69)	<0.001	0.65(0.47-0.76)	0.012
CD138+(low/moderate/high)	1.18(0.92-1.52)	0.195		
Tumour stroma percentage (low/high)	2.14(1.32-3.47)	0.002		0.197
Tumour budding (low/high)	2.51(1.56-4.04)	<0.001	1.91(1.14-3.19)	0.014
Ph-STAT1 tumour cell expression (low/moderate/high)	0.83(0.63-1.12)	0.230		
Ph–STAT3 tumour cell expression (low/moderate/high)	0.65(0.46-0.90)	0.011	0.69(0.51-0.95)	0.030
Locoregional treatment (lumpectomy+radiotherapy/ mastectomy +radiotherapy)	2.37(1.33-4.20)	0.003	2.03(1.19-3.74)	0.024
Systemic treatment (hormonal/hormonal+ chemotherapy/chemotherapy/ none)	1.11(0.86-1.43)	0.415		

**Figure 3:**
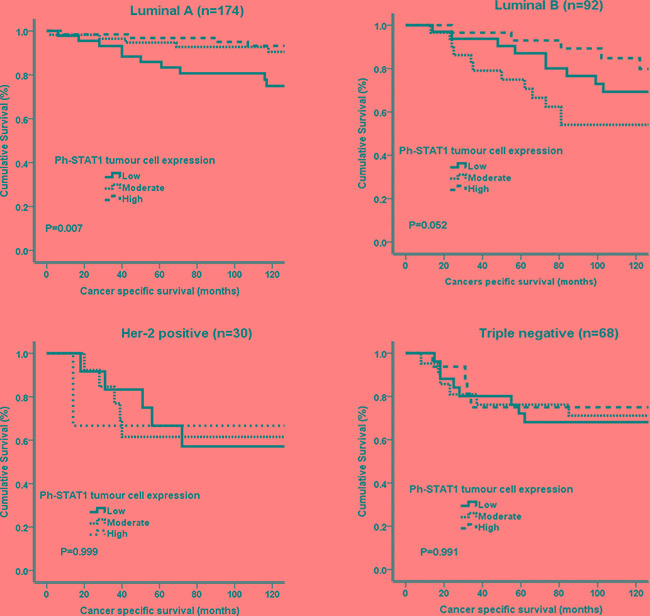
Kaplan-Meier survival curves (Log rank) of ph-STAT1 in different molecular subtypes Only Luminal A (n=174, 45%) shows significant association between high tumour cell expression of ph-STAT1 (n=121, 32%) and improved cancer specific survival

**Figure 4:**
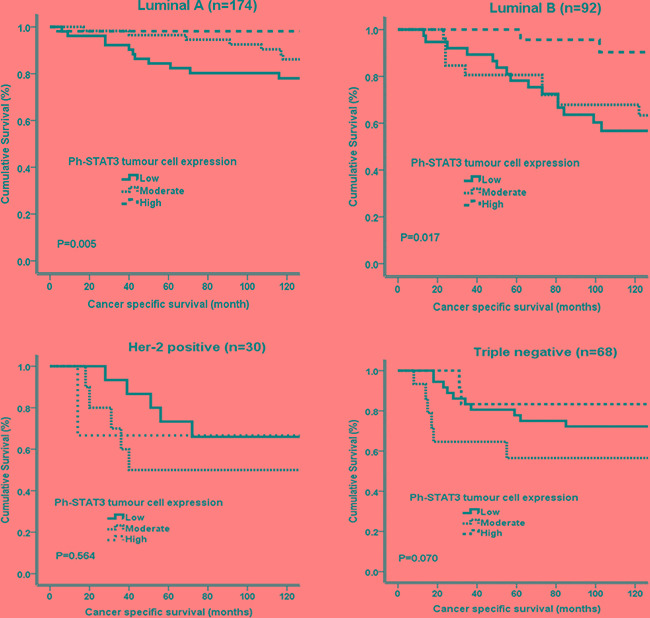
Kaplan-Meier survival curves (Log rank) of ph-STAT3 in different molecular subtypes Luminal A (n=174, 45%) and luminal B (n=92, 24%) show significant association between high tumour cell expression of ph-STAT3 (n=109, 28%) and improved cancer specific survival. Manipulation is permitted.

The relationship between ph-STAT1 and ph-STAT3 tumour cell expression and CSS using Kaplan-Meier log rank test, with relevance to different molecular subtypes, was examined (Figure 3 and 4). High ph-STAT1 was significantly associated with improved CSS in luminal A (n=174) tumours (*P*=0.007). High ph-STAT3 was significantly associated with improved CSS in luminal A (n=174) (*P*=0.005) and B (n=92) tumours (*P*=0.017). The small Her-2 positive subtype cohort (n=30) precluded meaningful analysis.

## DISCUSSION

In the present study, increased tumour cell expression of both ph-STAT1 and ph-STAT3 was associated with improved survival and the phenotypic characteristics of the tumour, in particular the low tumour grade and lack of tumour necrosis. Therefore, activation of tumour STATs may be an important mechanism by which the tumour cells mitigate the development of an aggressive phenotype in patients with invasive ductal breast cancer.

To our knowledge no previous study have conducted a comprehensive analysis of total and phosphorylated STAT1 and STAT3 expression in patients with ductal breast cancer. Ph-STAT1 and ph-STAT3 were strongly associated with each other independent of cellular location. In multivariate analysis, ph-STAT1 was independently associated with prolonged CSS, however when ph-STAT3 was also included in the model only ph-STAT3 remained independently associated with CSS. These results suggest that ph-STAT3 is the dominant STAT protein associated with improved survival in patients with invasive ductal breast cancer.

The observation that STAT1 is associated with improved survival may be explained by its role in promoting apoptosis and inhibition of proliferation [26]. STAT1 induces apoptosis by up-regulation of caspases 2 and 3 expression [27, 28] and recently Magou and colleagues have reported a positive association between ph-STAT1 and caspase 3 expression in primary breast cancer tissues [14]. Furthermore, STAT1 has been reported to inhibit the development of mammary tumours in experimental models [29, 30]. In certain contexts STAT3 also behaves as a tumour-suppressor protein targeting genes involved in apoptosis and induction of growth arrest [10, 31]. In particular, STAT3 is activated during the apoptotic involution of mammary gland [11, 12, 32] and the suppression of brain tumours [33]. Indeed, consistent with such a scheme, Sato and colleagues, a large dataset of more than 700 patients, reported that expression of ph-STAT3 was reduced in the progression from normal breast epithelia to invasive and metastatic breast cancer [19]. Furthermore, STAT3 has been shown to up-regulate tissue inhibitor of metalloproteinase-1 expression, which is recognised to reduce the invasiveness of breast cancer cells [34].

The results of the present study are consistent with the majority of previous reports where 6/10 studies reported that ph-STAT3 was associated with improved outcome (Table 1). Studies reporting no or poor prognosis in breast cancer were mainly performed in small cohorts, cohorts with limited follow-up, or had potential confounding factors. The present study was carried out in a cohort of ductal tumours with mature follow-up.

It is plausible that STAT1 and STAT3 have prognostic value in different tumour types, different molecular subtypes or in different aspects of the tumour microenvironment. In the present study, the prognostic role of ph-STAT1 and ph-STAT3 tumour cell expression in different molecular subtypes was examined. Ph-STAT1 and ph-STAT3 were significant predictors of prolonged CSS in luminal subtypes. Also, both ph-STAT1 and ph-STAT3 were directly associated with ER positive status. This may indicate that the role of STATs in breast cancer may be driven by endocrine hormones and support the cross-talk with ER [35, 36]. Previous reports have shown that patients with low proliferating luminal A tumours have higher ph-STAT3 expression compared to those with the luminal B tumours [37]. Furthermore, in ER negative, Her-2 positive tumours, no response was observed to trastuzumab in patients with STAT3 activation [38] and that JAK2 drives a JAK1/STAT3-independent signaling program in triple negative breast cancer [39], demonstrated that there are different activators and targets for STAT3 in different subgroups of breast cancer.

The present study reported an association between ph-STAT1, ph-STAT3 and the inflammatory cell infiltrate. High ph-STAT1 tumour cell expression was associated with up-regulation of local inflammatory infiltrate as evidenced by increased generalised inflammatory cell infiltrate. In contrast, high ph-STAT3 tumour cell expression was associated with down-regulation of the local inflammatory infiltrate as evidenced by decrease in the CD4+ T-lymphocytes. It was of interest that STAT1 and STAT3 were expressed in both the stroma fibroblasts and cells of the inflammatory infiltrate (Figure 1). Taken together, the results of the present study would suggest an important role for STAT1 and STAT3 in regulating anti-tumour immunity in the breast tumour microenvironment [40]. Such findings may be important in therapies to counteract immune dysfunction and improve cancer immunotherapy [9].

The present study reports for the first time a negative association between ph-STAT1 and ph-STAT3 expression and tumour necrosis. Moreover, that elevated ph-STAT3 expression was significantly associated with better survival, suggesting a protective role of STAT3 against tumour necrosis. The basis of such an observation is not clear, however it is of interest that IFNγ-induced STAT1 activation has been previously shown to negatively regulate hypoxia-inducible factor-1 (HIF-1) α-dependent transcription in human glioblastoma cells lines [41]. HIF-1 is a master regulator of the transcriptional response to hypoxia [42]. Indeed, constitutively active STAT3 acts as a master regulator of cell metabolism, inducing aerobic glycolysis via HIF-1 α transcriptional induction [43] as it is part of the complex signaling network that shapes the metabolic phenotype of tumour cells. Tumour hypoxia has been shown to be associated with a more clinically aggressive phenotype, resistance to therapy, angiogenesis and metastasis [44]. Therefore, further understanding of the molecular mechanism by which STAT1 down-regulates hypoxia-induced transcription may also lead to the development of a better therapeutic measure for cancer treatment.

Taken together, the results of the present study would suggest that both STAT1 and STAT3 act as tumour-suppressor proteins in patients with ductal breast cancer. STAT1 has long been implicated in growth suppression [26, 45] as loss of STAT1 function results in early development of breast tumours [29, 30]. Unlike other STAT members, the loss of STAT3 function results in early embryonic lethality STAT3 [46] and the suppression of tumour cell proliferation [47-49], suggesting its crucial role as an oncogenic factor. The mechanisms underlying STAT3 signalling pathway's diverse and sometimes opposing roles are still largely unknown. Nevertheless, it would suggest that the pleomorphic role of STAT3 in breast cancer prognosis, as an oncogene or a tumour suppressor, may be a function of the setting or cellular context, in particular the tumour microenvironment and necrosis. It may also suggest that there are other signal transduction pathways involved in the effect elaborated by tumour STAT3 expression. Irrespective, these results would indicate that STATs are central to the signaling networks in ductal breast cancer and that STAT3, in particular, has cross-talk with members of other pathways, such as the transcription factors HIF, and the nuclear factor kappa B [50, 51].

In the present study, although high ph-STAT1 and ph-STAT3 were associated with improved outcome, the clinical utility of targeting these pathways remains to be established. However, it is of interest that clinical trials evaluating Il-6/JAK/ STAT inhibitors in breast cancer patients are under way [25].

Potential limitations of the present study were that a specific examination of STAT1 and STAT3 in stromal tissue and in inflammatory cells was not carried since Chan and colleagues have reported that STAT1 expression may vary between tumour cells and the associated stroma, and have reported a selective loss of STAT1 expression in breast cancer cells but not in the surrounding stromal cells during tumour progression. Thus the increase in STAT1 levels in the subset of breast cancer cases that exhibit low STAT1 expression in the neoplastic cells could be explained by selective upregulation of STAT1 transcription in the stromal cells alone [30].

In conclusion, STAT1 and STAT3 tumour cell expression appears to be an important determinant of favourable outcome in patients with invasive ductal breast cancer. The present results suggest that STAT3 may affect disease outcome through the direct impact on tumour cells counteracting aggressive tumour features, as well as interaction with the surrounding microenvironment.

## MATERIALS AND METHODS

### Study materials

Patients presenting with invasive ductal breast cancer at Glasgow Royal Infirmary, Western Infirmary, and Stobhill Hospital, in the West of Scotland, between 1995 and 1998, and who had formalin-fixed paraffin embedded tissue blocks of the primary tumour available for evaluation were studied (n = 384). The study was approved by the Research Ethics Committee of the West Glasgow University Hospitals NHS Trust (REC reference is 07/s0704/61) and was performed according to the REMARK guidelines [52].

Clinicopathological data included age, tumour size, tumour grade, lymph node status, and type of surgery and use of adjuvant treatment (chemotherapy, hormonal therapy and/or radiotherapy) were retrieved from the routine reports. Tumour grade was assigned according to Nottingham Grading System. The ER and PR status were assessed on tissue microarrays (TMA) using immunohistochemistry with Dako ER antibody and Leica PR antibody, and scored according to the American Society of Clinical Oncology and College of American Pathologists guidelines with a cut-off value of 1% positive tumour nuclei [53]. Her-2 status was assessed on TMA as previously described i.e. a score 3+ is regarded as positive; 2+ is regarded as equivocal, leading to referral for Her-2 FISH; and 0 and 1+ are regarded as negative [54]. The molecular subtypes were defined as follows: Luminal A: oestrogen (ER) and/or progesterone receptor (PR) positive, Her-2 negative, low proliferative index (≤15%); Luminal B: hormone receptor positive, Her-2 positive, high proliferative index (>15%); Her-2 subtype: Her-2 positive and hormone receptor negative, any proliferative index; and Triple negative: Her-2 negative, hormone receptor negative, any proliferative index.

Lymph (LVI) and blood (BVI) vessel invasion were assessed, on 2.5 μm thick sections, using IHC staining with the lymphatic endothelial marker D2-40 (Covance, Monoclonal Antibody, SIG-3730, USA) diluted 1:100 and vascular endothelial marker Factor VIII (Mouse Monoclonal Antibody, NCL-L-Vwf, Leica, Newcastle, UK) diluted 1:100 as previously described [55].

Full-section haematoxylin and eosin (H&E) slides were used to score general local inflammatory infiltrate according to Klintrup-Mäkinen (KM) grade [56] and tumour necrosis as previously described [57]. Briefly, tumours were scored on four-point scores based on appearances at the tumour invasive margin. A score of 0 signified that there were no inflammatory cells at the tumour's invasive margin; score 1 indicated mild and patchy inflammatory cells; score 2 denoted a prominent band-like inflammatory reaction at the invasive margin; and score 3 revealed a florid cup-like inflammatory infiltrate at the invasive edge. The extent of necrosis was assessed at high power as absent (only single-cell death identifiable); mild (necrosis in <25% of fields); moderate (necrosis in 25-50% of fields) and extensive (confluent necrosis in >50% of fields) which then grouped into low and high grade.

Individual immune cells infiltrate was assessed using IHC staining on TMA sections for macrophages, helper and cytotoxic T-lymphocytes, and plasma cells using CD68, CD4, CD8 and CD138 antibodies respectively, as previously described [58].

Full-section H&E slides were also used to score the tumour stroma percentage (TSP) and tumour budding as previously reported [59-61]. Briefly, at ×5 magnification, an area representative of the tumour invasive margin was selected, and then single field of ×10 magnification was examined, ensuring that tumour cells were present at all four sides of the image and the area of stroma was calculated as a percentage; low grade TSP (≤50%) or high grade (>50%). For budding, an area representative of the tumour invasive margin was selected at ×5 magnification, and a grid of 0.385mm^2^ size was drawn at five highest budding areas. The highest bud count per field was used as the number of buds.

### Immunohistochemistry of STAT1 and STAT3

Immunohistochemical expression of total STAT1, Y701 phosphorylated STAT1 (ph-STAT1) total STAT3 and Y705 phosphorylated STAT3 (ph-STAT3) were carried out using a previously constructed TMA. Sections of 2.5 μm thickness from each TMA block were placed on silanized glass slides. Sections were dewaxed in xylene before being rehydrated using graded alcohols. Antigen retrieval for all STATs isoforms was performed using Tris-EDETA buffer (pH 8) for 20 minutes before cooling for 20 minutes. Endogenous peroxidase activity was blocked using 3% hydrogen peroxide for 20 minutes before rinsing in water. Normal horse serum at dilution 1:10 was applied for 30 minutes at room temperature as a blocking solution. TMA sections were then incubated overnight at 4°C with the primary antibodies as following: total STAT1 (STAT1 (42H3) Rabbit monoclonal antibody, code 9175, Cell Signaling Technology, USA) at a concentration of 1:100; ph-STAT1 (Rabbit PAb to STAT1 phosphoY701, code ab30645, Abcam, Cambridge) at a concentration of 1:150; total STAT3 (STAT3 Rabbit Ab, code 9132L, Cell Signaling Technology, USA) at a concentration of 1:200; Ph-STAT3 (Y705) antibody (P-STAT3 (Y705) Rabbit Ab, code 9131L, Cell Signaling Technology, USA) at a concentration of 1:200. Sections were then washed in TBS for ten minutes. Envision (Dako) was then added to the sections for 30 minutes at room temperature before washing in TBS for ten minutes. DAB substrate was added for five minutes until colour developed before washing in running water for ten minutes. Slides were then counterstained in haematoxylin for 60 seconds and blued with Scotts’ tap water before being dehydrated through a series of graded alcohols. Cover slips were applied using distrene, plasticizer, xylene (DPX). Negative and positive controls were included in the staining runs.

### Slide scanning and scoring

Stained TMA sections were scanned using a Hamamatsu NanoZoomer (Welwyn Garden City, Hertfordshire, UK) at x20 magnification and visualization was carried out using Slidepath Digital Image Hub, version 4.0.1 (Slidepath, Leica Biosystems, Milton Keynes, UK). Assessment of total STAT1, ph-STAT1, total STAT3 and ph-STAT3 expression within the cancer cell cytoplasm and nucleus was performed by a single examiner (FJG) blinded to clinical data at x20 magnification (total magnification x40) using the weighted histoscore. The weighted histoscore provides an assessment of the percentage and density of staining and is calculated as follows: 0x% not stained + 1x% weakly stained + 2x % moderately stained + 3x % strongly stained. This gives a range of scores from 0 to 300. Total and ph-STATs expression within the cytoplasm and nucleus were calculated separately. To ensure reproducibility of scoring, 15% of tumours for each antibody was co-scored by a second investigator (J.E.) blinded to other data. The intraclass correlation coefficient (ICCC) was 0.852 and 0.831 for cytoplasmic and nuclear total STAT1 respectively, and 0.797 and 0.871 for cytoplasmic and nuclear ph-STAT1 respectively. The ICCC was 0.795 and 0.801 for cytoplasmic and nuclear total STAT3 respectively, and 0.814 and 0.782 for cytoplasmic and nuclear ph-STAT3 respectively, indicating good agreement.

### Statistical analysis

For the purpose of statistical analysis, patients were split into two groups on the basis of the mean value of cytoplasmic and nuclear STAT1/STAT3 weighted histoscore, as low cytoplasmic and low nuclear STAT1/STAT3 expression and high cytoplasmic or high nuclear STAT1/STAT3 expression. In order to identify the impact of cellular STAT1/STAT3 expression at both cytoplasmic and nuclear location, an expression code was developed (STAT1/STAT3 tumour cell expression) as follows: patients with both low cytoplasmic and nuclear expression were classified as the low tumour cell expression group, patients with either cytoplasmic or nuclear expression is low were classified as the moderate tumour cell expression group, and patients with both high cytoplasmic and high nuclear expression were classified as the high tumour cell expression group. These analyses have been applied for total and for ph-STAT1 separately and total and ph-STAT3 separately.

Subsequently, the relationship between clinicopathological characteristics, ph-STAT1 tumour cell expression and ph-STAT3 tumour cell expression were examined using the Chi-square test for linear trend. The relationship between total and ph-STAT1 tumour cell expression, total and ph-STAT3 tumour cell expression and cancer-specific survival was examined using Kaplan-Meier log-rank analysis. Univariate survival analysis was performed using Cox proportional hazards regression. Variables with *P*-value of <0.1 were entered into a multivariable model using a backwards conditional method. A *P*-value <0.05 was considered statistically significant. All analyses were performed using SPSS version 22.0 (IBM SPSS).

Patients were routinely followed-up following surgery. Date and cause of death was cross-checked with the cancer registration system and the Registrar General (Scotland). Death records were complete until 31st of May 2013 and that served as the censor date. Cancer-specific survival was measured from the date of primary surgery until the date of death from breast cancer.
